# Removal of a Maxillary Third Molar Displaced into Pterygopalatine Fossa via Intraoral Approach

**DOI:** 10.1155/2013/392148

**Published:** 2013-02-07

**Authors:** Nedim Özer, Fulya Üçem, Alp Saruhanoğlu, Serdar Yilmaz, Hakkı Tanyeri

**Affiliations:** ^1^Department of Oral and Maxillofacial Surgery, Faculty of Dentistry, Istanbul Medipol University, Turkey; ^2^Department of Oral and Maxillofacial Surgery, Faculty of Dentistry, Istanbul University, Istanbul, Turkey

## Abstract

The removal of impacted maxillary third molars is one of the most common procedures performed in oral and maxillofacial surgery units with low rates of complications and morbidity. A few cases of accidental displacement of third molars into adjacent anatomical spaces, such as the infratemporal fossa, the pterygomandibular space, the maxillary sinus, buccal space, or the lateral pharyngeal space, during surgical interventions have been reported. In this paper, a case of a maxillary third molar accidentally displaced into the pterygopalatine fossa is presented, and the removal of the tooth via intraoral approach is described.

## 1. Introduction

The removal of impacted maxillary third molars is one of the most common procedures performed in oral and maxillofacial surgery units with low rates of complications and morbidity [1–3]. Most frequently confronted complications are fracture of tuberosity, tooth root fracture, perforation of the maxillary sinus, prolapse of the buccal fat pad, and displacement of the roots or tooth into the maxillary sinus [3–5]. According to the literature, a few cases of accidental displacement of molars into adjacent anatomical spaces, such as the infratemporal fossa, the pterygomandibular space, the maxillary sinus, the buccal space, or the lateral pharyngeal space, during surgical interventions have been reported [3, 5, 6]. However this is the first reported case of maxillary third molar displaced into pterygopalatine fossa. The aim of this case report is to identify potential risk factors and to gather information on the prevention and treatment of this complication.

## 2. Case Report

**Figure 1:**
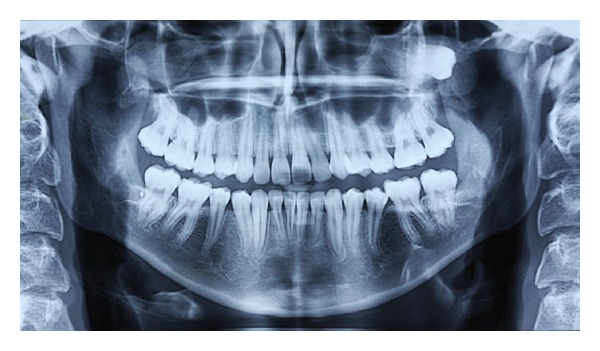
A panoramic radiograph revealed that the left maxillary third molar was displaced in a posterosuperior direction.

**Figure 2:**
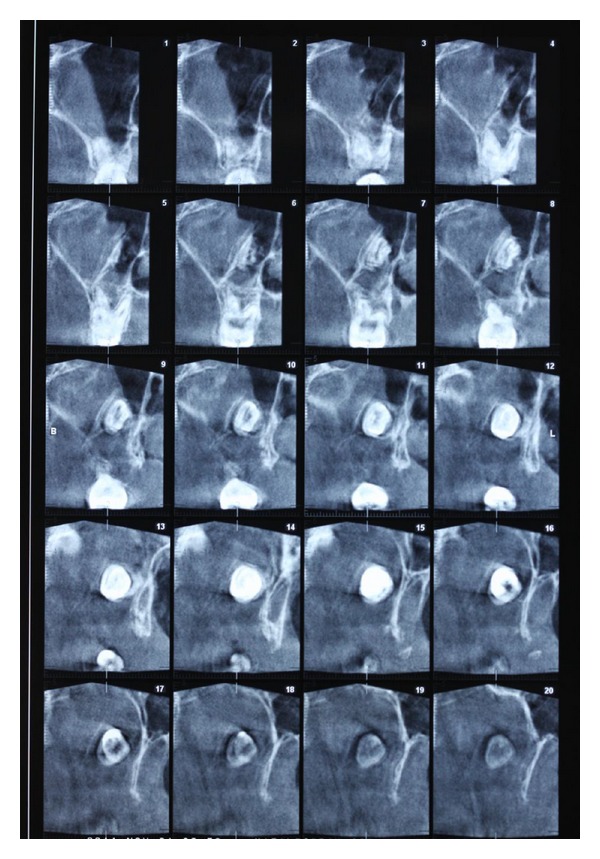
CT revealed that the tooth was located superiorly between the distal margin of maxillary tuberosity and the anterior border of lateral pterygoid plate.

**Figure 3:**
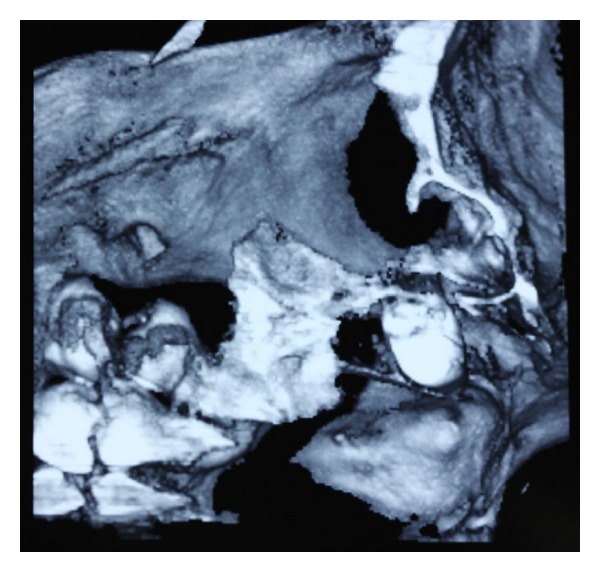
CT revealed that the root was stuck in the antrum of pterygopalatine fossa.

A 23-year-old girl was referred to our clinic for the assessment of a maxillary left third molar displacement that occurred during surgery performed 1 week earlier. The patient had slight facial swelling and restricted mouth opening. Intraoral examination revealed that the dislodged third molar was not palpable within the soft tissues. Immediately, a panoramic radiograph was taken which revealed that the left maxillary third molar was displaced in a posterior direction possibly in the infratemporal fossa area (Figure 1). For being able to determine the precise position of the tooth, computed tomography (CT) scans were taken. CT revealed that the tooth was located superiorly between the distal margin of maxillary tuberosity and anterior border of lateral pterygoid plate; the root was placed in the antrum of pterygopalatine fossa (Figures 2 and 3). The general anesthesia was the surgeon's choice due to limited working area and patient's psychologic unease that may cause further displacement. The classical maxillary third molar surgery flap design was performed as vertical incision mesial to the first molar and horizontal incision extended to the distal margin of the maxillary tuberosity. Mucoperiosteal flap was reflected. Upon the reflection of the flap the pathway of the displaced third molar has been revealed as the posterior aspect of maxillary sinus area was open to site. Extending through the posterior wall of maxillary sinus and with careful exploring the tooth was reached and exposed with a straight elevator. The granulation tissue around the tooth was removed and the tooth was extracted. Maxillary sinus and deep layers were irrigated with %0.5 saline solution and with Rifocin. The incision was primarily closed. No complication occurred postoperatively after 1 year of followup.

## 3. Discussion

Displacement of maxillary third molars into the neighbouring anatomic spaces is associated with insufficient clinical and radiographic examination, lack of basic principles of surgery such as poor anatomic knowledge, inadequate flap, decreased visibility, and excessive or uncontrolled force applied during extraction [3–6].

Maxillary third molars uncommonly displaced through the periosteum into the infratemporal fossa just adjacent to the lateral pterygoid plate and inferior to the lateral pterygoid muscle [7]. Excessive force application and incorrect use of elevator during the attempt to retrieve the tooth may further displace the tooth upward into the skull base carrying greater risks for morbidity [6, 7]. 

The access for the surgical removal of the tooth from pterygopalatine fossa is very difficult and has the potential for morbidity because it encloses vital anatomic structures. The pterygopalatine fossa is a complex anatomic structure which has the shape of an inverted cone [8]. It is located lateral to the nasal cavity, anterior/inferior to the middle cranial fossa, inferior to the apex of the orbit, and medial to the infratemporal fossa. The pterygopalatine fossa contains maxillary nerve (second branch of the trigeminal nerve), Vidian nerve, the pterygopalatine ganglion, and the third part of the maxillary artery [8].

Because the exact localization of the displaced tooth is impossible to determine clinically, radiographic examination is indicated [4, 7]. The superimposition of the anatomic structures located at the site of the infratemporal and pterygopalatine fossa may disorient the diagnosis in the case [3–5, 7]. So as to allow to determine the precise and detailed location of the dislodged tooth computed tomography examination is needed [3, 7].

The removal time of the displaced tooth is controversial in the literature [4]. Some authors propose to deliver the displaced tooth immediately because of the risks of infection, foreign body reaction and because of its anatomic location which can have the potential for morbidity [3, 7]. According to some authors, displaced teeth can migrate downwards into the oral cavity, allowing an easy surgical removal [4, 7]. Nonetheless according to others, migration of the tooth is impossible because of fibrosis and anatomical boundaries [3, 4, 7].

In our case, the patient had pain and restricted mouth opening. The cone beam volumetric tomography scan showed clearly that the displaced tooth was just barely stuck inside the pterygopalatine fossa. Because the pterygopalatine fossa encloses vital structures, further displacement could have potential symptoms associated with involvement of the neurovascular structures and pterygopalatine muscles, such as trismus, lateral pharyngeal swelling, hypoesthesia, proptosis, diplopia, pain, and nasal obstruction [9, 10]. Due to being dislodged into the pterygopalatine fossa area, immediate surgery was planned as the patient was referred to our clinic, so as to prevent damage and further complication risks.

Many surgical approaches have been used for the retrieval surgery of displaced maxillary third molar into the infratemporal fossa area such as long incision in the buccal sulcus, Gillies's approach, the Caldwell-Luc approach through the maxillary sinus after removal of the whole posterior wall, and resection of the coronoid process [1–3, 6]. 

In our case conservative method of surgery via intraoral approach was preferred due to the third molar location, being stuck in the antrum of pterygopalatine fossa. A long incision extending distal to the maxillary tuberosity and a blunt dissection behind the maxillary sinus wall were performed until reaching the tooth. To prevent further dislocation a retractor was placed distal area of the tooth. A soft pressure applied with an elevator, and the tooth was extracted.

As a result if a complication does occur during third molar extraction such in our case, dentists should immediately refer the patient to an oral and maxillofacial surgeon and should not try to remove the displaced root without proper assurance. This is imperative for being able to evaluate the condition of the tooth preoperatively, select adequate instruments and technique, and take good care during extraction and prevent the risk of hemorrhage, neurologic injury, and further displacement of the tooth. Localization with images and proper surgical methods are the keys to retrieving the displaced fragment successfully. There are no certain treatment choices whether immediate or secondary surgery is advantageous for the retrieval of such displaced teeth. The oral and maxillofacial surgeon decides uniquely evaluating the time the patient was referred, location of the tooth, and the patient's psychological conditions all together for the most appropriate surgery approach.
